# Effects of Fullerenol Nanoparticles on Rat Oocyte Meiosis Resumption

**DOI:** 10.3390/ijms19030699

**Published:** 2018-03-01

**Authors:** Runhong Lei, Xue Bai, Yanan Chang, Juan Li, Yanxia Qin, Kui Chen, Weihong Gu, Shibo Xia, Jiaxin Zhang, Zhenbo Wang, Gengmei Xing

**Affiliations:** 1 CAS Key Laboratory for Biomedical Effects of Nanomaterials and Nanosafety, Institute of High Energy Physics, Chinese Academy of Sciences, Beijing 100049, China; leirh@ihep.ac.cn (R.L.); xbai@ihep.ac.cn (X.B.); changyn@ihep.ac.cn (Y.C.); lijuan@ihep.ac.cn (J.L.); qinyx@ihep.ac.cn (Y.Q.); chenkui@ihep.ac.cn (K.C.); guwh@ihep.ac.cn (W.G.); xiasb@ihep.ac.cn (S.X.); zhangjiaxin@ihep.ac.cn (J.Z.); 2 State Key Laboratory of Stem Cell and Reproductive Biology, Institute of Zoology, Chinese Academy of Sciences, Beijing 100101, China

**Keywords:** fullerenol, oocyte meiosis resumption, gap junction, granulosa cells, EGFR/ERK1/2

## Abstract

The excellent biocompatibility and biological effects of fullerenol and its derivatives make their biomedical application promising. The potential effects of fullerenol in mammals have been extensively studied, but little is known about its effects on female reproduction. Using canonical oocyte-granulosa cell complexes (OGCs) in vitro maturation culture model, we investigated the effect of fullerenol on the first oocyte meiotic resumption. In the surrounding granulosa cells, fullerenol nanoparticles occluded the extracellular domain of the epidermal growth factor receptor (EGFR) to reduce EGFR-ligand binding and subsequent extracellular signal-regulated kinase 1 and 2 (ERK1/2) activation, which involved the regulation of connexin 43 (CX43) expression and internalization. Downregulation of CX43 expression and the retraction of transzonal projections (TZPs) interrupted the gap junction channel and TZPs based mass transportation. This effect decreased cyclic adenosine monophosphate (cAMP) levels in the oocyte and thereby accelerated rat oocyte meiosis resumption. Moreover, perinuclear distribution of CX43 and EGFR was observed in granulosa cells, which could further exacerbate the effects. Fullerenol nanoparticles interfered with the strict process of oocyte meiosis resumption, which likely reduced the oocyte quality.

## 1. Introduction

Polyhydroxylated fullerenols are typical carbon nanoparticles that have excellent water solubility and superior biocompatibility characteristics. They are considered one of the most promising diagnostic, therapeutic nanoparticles, and even as a protective agent against cancer [1,2,3,4,5]. Necessary concerns over the potential adverse effects of these nanoparticles on human health have been raised. Numerous experiments have ascertained some biological activities of the nanoparticles [6,7,8]. Women are relatively more vulnerable to the potential hazardous effect and need to be especially cautious because the adverse effect of nanoparticles on this population could affect the reproductive ability and fetal development.

Studies have extensively investigated the effects of nanoparticles on mammalian reproduction including gold, silver, gold and silver alloys [9,10,11,12], selenium [13], zinc oxide [14], nickel [15], graphene oxide [16,17] and titanium dioxide [18,19]. In contrast to the considerable investigations of these nanoparticles, the effect of fullerenol on reproduction has not been widely evaluated. Fullerenol has been reported to eliminate superoxide anion free radical, which could suppress oxidative stress to protect spermatozoa [20,21], and reduce oxidative stress induced by drugs transported into ovary cell lines in vitro [22]. The reproductive and developmental toxicology of carbon-based nanomaterials was extensively reviewed by Ema et al. [23]. However, the effect of fullerenol nanoparticles on female reproduction is still unclear.

Evidence indicates that small size nanoparticles could accumulate in the uterine wall when administered intravenously to rats and mice [24,25]. In vitro studies indicate that oocyte maturation was reduced when prenatal follicles were exposed to titanium dioxide [26]. These results suggest that nanoparticles can penetrate follicles. In our preliminary experiment, pre-administration of fullerenol via the female rat tail vein impaired fetal growth (detailed in the Materials and Methods, and Figure S1 in the Supplementary Materials), which similarly suggests their permeation and potential negative effects. Consequently, the potential effect of nanoparticles, including fullerenol, should be carefully and comprehensively investigated.

In mammals, poor quality eggs are the main factor increasing the risk of infertility, spontaneous abortion, and congenital birth defects [27]. Primordial oocytes are generated at the fetal stage. Before maturation and ovulation, oocytes are arrested at the first meiosis, indicated by the presence of the germinal vesicle (GV). A surge of luteinizing hormone (LH) or the artificial removal of the oocytes from follicles decreases cyclic adenosine monophosphate (cAMP) concentrations in oocyte to induce germinal vesicle breakdown (GVBD), which symbolizes the oocyte meiotic resumption [28,29]. Poor egg quality is largely due to unfavorable oocyte maturation [27]. In follicles, granulosa cells connect to each other and surround the oocyte to control its maturation. Connexin 43 (CX43)-based gap junctions between the surrounding granulosa cells are formed. Moreover, transzonal projections (TZPs) are generated from the innermost layer of the granulosa cells to cross the zona pellucida and target oocytes. CX43 on the terminal of the projections docks with CX37 in oocytes, forming heterotypic junctions between the oocyte and granulosa cells [30,31]. Both the gap junctions and TZPs play essential roles in fully acquiring developmental potency by controlling oocyte arrest and meiotic resumption through ensuring the transport of essential elements, which guarantee oocyte maturation [32,33]. Loss of gap junction and retraction of TZPs occur before the initiation of GVBD [34,35,36]. However, premature GVBD is one of the causes of reduced oocyte quality and female fertility [37]. Oocyte quality is a key factor in determining embryo development [38]. Granulosa cell-controlled oocyte meiosis resumption has a critical role in the acquisition of development competence [39,40]. Therefore, we speculated that fullerenol could penetrate follicles and affect oocyte meiosis resumption.

In this study, we used an oocyte-granulosa cell complexes (OGCs) in vitro maturation culture model, which is widely used in female reproductive biology and toxicology studies [41], to investigate the potential effect of fullerenol on the initiation of oocyte meiotic resumption. Our findings indicate that fullerenol nanoparticles interfered with the strict programmed initiation of oocyte meiotic resumption by occluding the extracellular domain of the epidermal growth factor receptor (EGFR) to inhibit EGFR-ligand binding and induce CX43 and EGFR perinuclear distribution. Our data provide an important molecular basis for the further elucidation of the effects of fullerenol nanoparticles on organisms.

## 2. Results

### 2.1. Fullerenol Accelerated Transzonal Projection (TZP) Retraction

**Figure 1:**
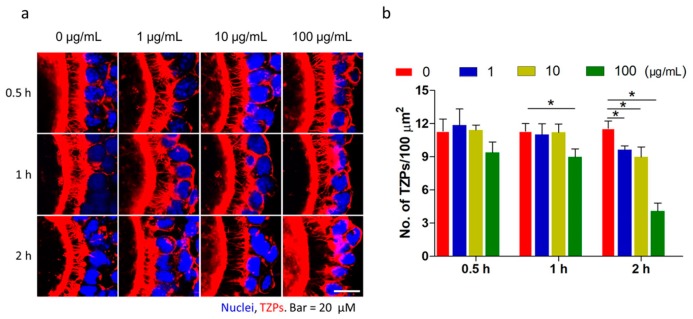
Fullerenol accelerates transzonal projection (TZP) retraction. Freshly isolated oocyte-granulosa cell complexes (OGCs) were cultured in medium with different concentrations of fullerenol (0, 1, 10, and 100 μg/mL) for different times (0.5, 1, and 2 h). TZP number and integrity were evaluated using fluorescent labeling. (**a**) Representative images of the F-actin-rich TZPs between oocyte and surrounding granulosa cells are shown (red, scale bar: 20 μm). Blue color indicates granulosa cell nuclei; (**b**) Intact TZPs were counted and expressed as mean ± SEM (*n* = 5–6). Experiment was performed three times, * *p* < 0.05.

TZPs were observed in OGCs incubated with fullerenol (0, 1, 10, and 100 μg/mL) for different durations (0.5, 1, and 2 h). The confocal microscopy revealed intact and abundant TZPs, which mainly consisted of F-actin and were labeled with rhodamine phalloidine and were clearly visible as thin filaments connecting the granulosa cell layer and oocytes. After treatment, the number of TZPs was clearly reduced in a time- and dose-dependent manner (Figure 1a,b). Then, 2 h later, a few thin filaments from granulosa cells connecting oocyte were observed in the 100 μg/mL fullerenol group, but they were still intact and abundant in the control group. The number of TZPs was reduced in cells exposed to 1 and 10 μg/mL fullerenol nanoparticles. These results demonstrated that fullerenol accelerated TZP retraction from the oocytes.

### 2.2. Fullerenol Decreased Connexin 43 (CX43) Expression in Granulosa Cells

**Figure 2:**
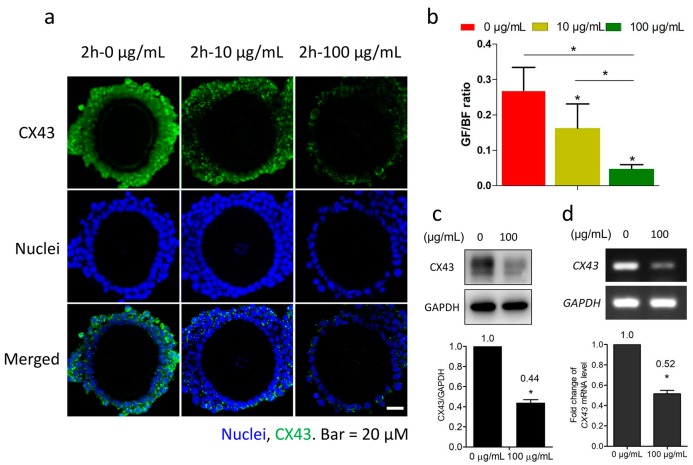
Fullerenol downregulates connexin 43 (CX43) expression. Oocyte-granulosa cell complexes (OGCs) were cultured in medium with different concentrations of fullerenol (0, 10, and 100 μg/mL) for 2 h, and then fixed for immunofluorescent staining or harvested after removal of oocytes for immunoblotting and polymerase chain reaction (PCR) analysis. (**a**) Reduced CX43 protein levels in granulosa cells. Green, CX43, Blue, nuclei; scale bar: 20 μm; (**b**) Quantitative analysis of CX43 fluorescence intensity normalized to nuclear fluorescence intensity. Data are mean ± standard error of the mean (SEM, *n* = 6–9 OGCs); (**c**) Immunoblotting detection of CX43 protein and (**d**) mRNA levels in granulosa cells after 2 h fullerenol treatment. Data are mean ± standard deviation (SD). At least three individual experiments were performed. The mean value was presented in the quantitative image. GF, green fluorescence intensity (CX43). BF, blue fluorescence intensity (nuclei). Each experiment was performed at least three times with similar results; * *p* < 0.05 compared with the indicated group.

CX43 is one of the major connexins in connected granulosa cells. Following 2 h culture in M2 medium with fullerenol (10 and 100 μg/mL), antibody-labeled CX43 was visible in granulosa cells under a fluorescent confocal microscope. As shown in Figure 2a, the CX43 (green) intensity in peripheral granulosa cells of the OGCs was reduced in a dose-dependent manner. The relative fluorescent intensity analysis of labeled CX43 (green) and the nuclei (blue) revealed a significant difference between the compared groups, even the 10 μg/mL group (Figure 2b). The CX43 expression in granulosa cells treated with 100 μg/mL fullerenol was downregulated by approximately 56% according to the western blot analysis (Figure 2c). Furthermore, transcriptional levels of CX43 were also downregulated (Figure 2d).

### 2.3. Fullerenol Induced Perinuclear Distribution of CX43 in Granulosa Cells

**Figure 3:**
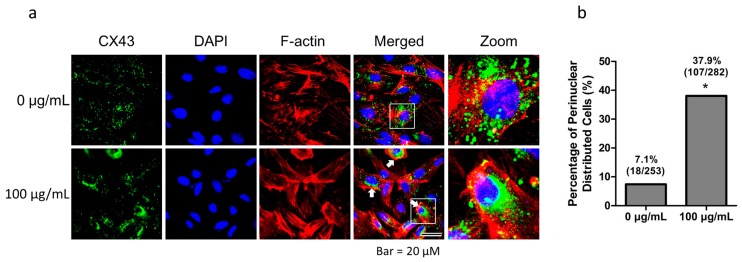
Fullerenol induces connexin 43 (CX43) perinuclear distribution. Freshly isolated granulosa cells were routinely cultured for 4 days for attachment and were then treated with 100 μg/mL fullerenol for 2 h. Cellular distribution of CX43 was evaluated. (**a**) Immunofluorescent analysis of CX43 (green, scale bar: 20 μm). Fullerenol treatment caused CX43 perinuclear distribution compared with the control group (indicated by white arrow). The white squares indicate the distinctive cells and are zoomed. Blue and red color indicate nuclear staining and F-actin skeleton, respectively; (**b**) Percentage of granulosa cells with CX43 perinuclear distribution; * indicates the test statistic was greater than the critical value when the two percentages were compared.

We examined the CX43 distribution in granulosa cells. After collection, granulosa cells were routinely cultured for 4 days, and their purity was confirmed using the specific marker follicle stimulating hormone receptor (FSHR) (Figure S2). The adherent granulosa cells were treated with 100 μg/mL fullerenol for 2 h, and then CX43 was labeled with the specific antibody. The fullerenol-treated cells showed CX43 (green) distribution in the perinuclear region, which was mostly expressed in the cytoplasm and appeared as aggregate spots in the control cells (Figure 3a). The percentage of cells with a perinuclear distribution of CX43 was 37.9% (107/282) and only 7.1% (18/253) in the treated and control groups, respectively (Figure 3b).

### 2.4. Fullerenol Expedited cAMP Reduction in Oocyte and Induced GVBD

**Figure 4:**
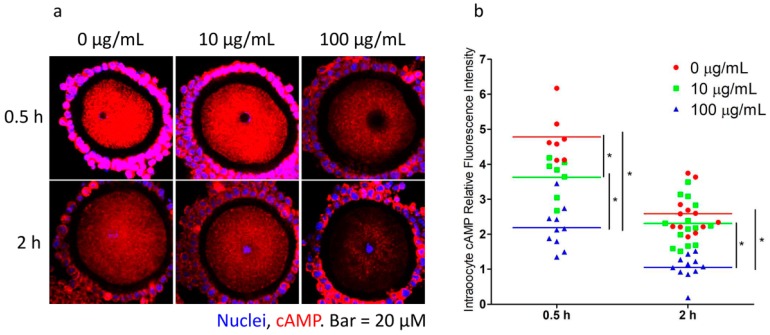
Immunofluorescent analysis of cyclic adenosine monophosphate (cAMP) in oocytes (red, scale bar: 20 μm). Oocyte-granulosa cell complexes (OGCs) were cultured in M2 medium with different concentrations of fullerenol (0, 10, and 100 μg/mL) for 0.5 and 2 h and then fixed. (**a**) Representative images of cAMP in OGCs; (**b**) Quantitative analysis of intraoocyte cAMP fluorescence intensity (*n* = 7–14 oocytes). Blue and red color indicate nuclei staining and cAMP, respectively. Experiment was performed three times with similar results; * *p* < 0.05 compared with the indicated groups.

The gap junction and TZPs provide crucial channels for accumulating cAMP and other essential components from granulosa cells to the oocytes. The accelerated retraction of TZPs could decrease the cAMP levels in oocytes, leading to GVBD. OGCs were cultured in M2 medium containing fullerenol (0, 10, and 100 μg/mL) for 0.5 and 2 h and then they were quickly fixed, followed by labeling of the cAMP content in the oocytes. Fullerenol reduced the cAMP (red) content in oocyte in a dose-dependent manner. At a concentration of 10 μg/mL and the 0.5 h time point, significant cAMP reduce was observed and the lowest level was observed in the 100 μg/mL group after a 2-h treatment (Figure 4).

Then, the measurement of GVBD rates of the oocyte showed that it significantly increased following a 5-h treatment with fullerenol compared with the control (Table 1). The rate increased from 80.3% (212/264) in the control to 87.7% (186/212), 89.8% (239/266), and 90.0% (162/180), in the 1, 10, and 100 μg/mL fullerenol-treated groups, respectively. Particularly, 1 μg/mL fullerenol increased the GVBD rate.

### 2.5. Fullerenol Inhibited Extracellular Signal-Regulated Kinase 1 and 2 (ERK1/2) Activity by Occluding Ligand-Epidermal Growth Factor Receptor (EGFR) Binding

**Figure 5:**
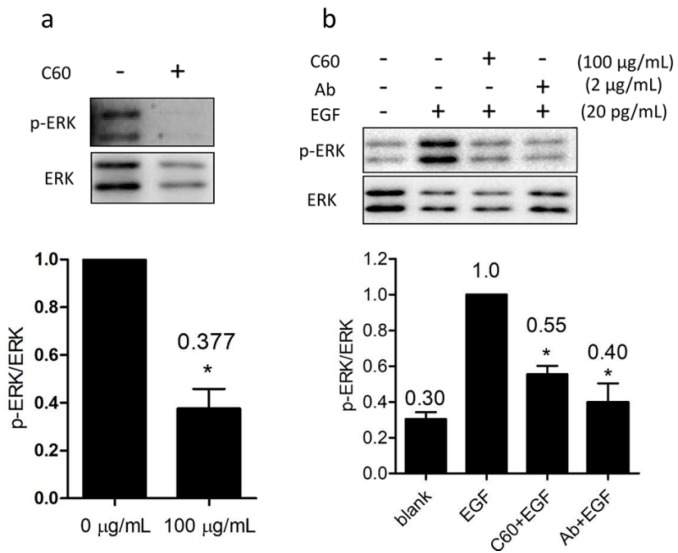
Fullerenol reduces extracellular signal-regulated kinase 1 and 2 (ERK1/2) activation by blocking epidermal growth factor receptors (EGFRs). Freshly isolated oocyte-granulosa cell complexes (OGCs) were cultured in M2 medium with 100 μg/mL fullerenol (presented as C60) and 2 μg/mL EGFR antibody (presented as Ab) for 2 h, and then granulosa cells were harvested after removing oocytes following continuous stimulation with or without EGF (20 pg/mL, 10 min). (**a**) Fullerenol treatment (100 μg/mL) significantly reduced ERK1/2 activation in granulosa cells; (**b**) Fullerenol or extracellular domain targeted anti-EGFR antibody pretreatment (100 μg/mL for 2 h) reduced EGF-induced-ERK1/2 activation. Mean values are presented in the quantitative image. Ab, extracellular domain targeted anti-EGFR antibody. At least three individual experiments were performed. Data are shown as mean ± standard deviation (SD); * *p* < 0.05 compared with the control.

EGFR-mediated ERK1/2 activation is one of the regulatory mechanisms mediating CX43 expression [42]. Using western blot, we found that 2 h treatment with 100 μg/mL fullerenol significantly decreased the p-ERK1/2 level (presented as p-ERK in Figure 5), by approximately 62% in granulosa cells compared with the control (Figure 5a). Ligand binding to EGFR is an essential step for ERK1/2 activation. EGF, an EGFR ligand, efficiently activated ERK1/2 while binding of the anti-EGFR antibody to the extracellular domain of EGFR inhibited EGFR/ERK1/2 activation by occluding ligand binding [43]. Therefore, the antibody (Ab in Figure 5) against EGFR was selected as a positive control, and it significantly reduced p-ERK1/2 levels (Figure S3). Granulosa cells were pretreated with 100 μg/mL fullerenol or 2 μg/mL antibody for 2 h and then stimulated with EGF to activate EGFRs. Western blot showed a significant reduction in p-ERK1/2 levels in fullerenol- and antibody-pretreated granulosa cells compared with the untreated group (Figure 5b). Analysis of these results showed that fullerenol apparently also inhibited EGFR-mediated ERK1/2 phosphorylation by occluding the ligand-binding region (Figure 5b).

### 2.6. Fullerenol Reduced Extracellular Domain-Targeted Anti-EGFR Antibody Binding

**Figure 6:**
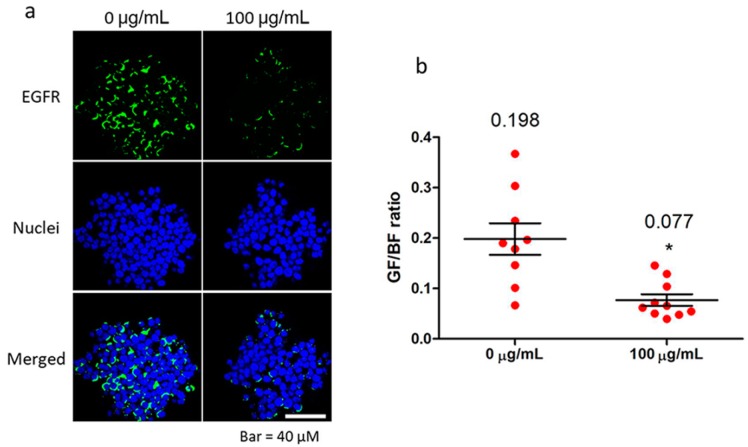
Fullerenol reduces extracellular domain targeted anti-epidermal growth factor receptor (EGFR) antibody binding. Freshly isolated oocyte-granulosa cell complexes (OGCs) were quickly fixed for immunofluorescent staining. During the standard procedure, OGCs were exposed to 100 μg/mL fullerenol for 10 min, and then gently washed before incubation with primary antibody. (**a**) Representative images of EGFR immunofluorescently labeled with an extracellular domain-targeted antibody; (**b**) Relative quantitative analysis of EGFR fluorescence intensity (*n* = 9–10 views) normalized to fluorescence intensity of nuclei. Green and blue indicate EGRF and nuclei, respectively. GF, green fluorescence intensity (EGFR). BF, blue fluorescence intensity (nuclei). Scale bar: 40 μm. Mean values are presented in quantitative image. Data are mean ± standard error of the mean (SEM); * *p* < 0.05 compared with the control.

Then, we investigated whether fullerenol directly occludes the EGFR extracellular domain to reduce the targeted antibody binding using immunofluorescent labeling. Compared with the control group, an obvious reduction of detectable EGFR antibody (green) targeting EGFR in granulosa cells was found after treatment with 100 μg/mL fullerenol for 10 min (Figure 6a). Quantitative analysis of the fluorescence intensity indicated that the reduction was approximately 61% (Figure 6b). This indicated that fullerenol directly occluded the extracellular domain of EGFR to inhibit targeted ligand binding.

### 2.7. Fullerenol Altered EGFR Distribution in Granulosa Cells

**Figure 7:**
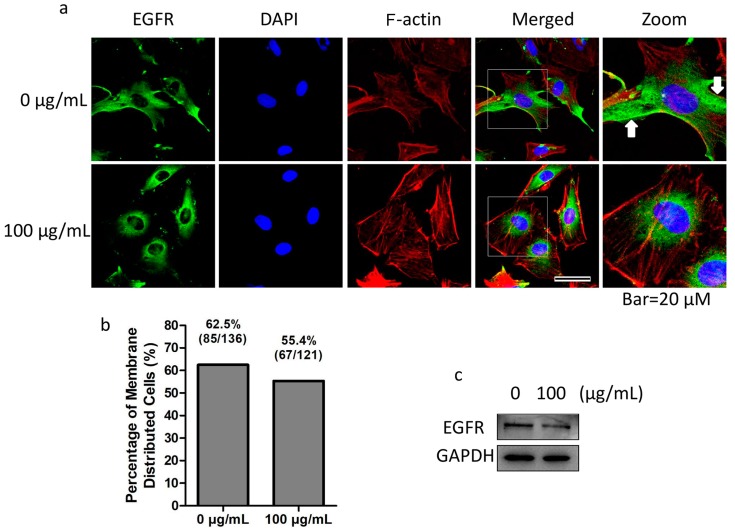
Fullerenol changes distribution pattern of epidermal growth factor receptor (EGFR). Adherent granulosa cells or oocyte-granulosa cell complexes (OGCs) were treated with or without 100 μg/mL fullerenol for 2 h and then harvested or fixed. (**a**) Fullerenol treatment reduced EGFR membrane distribution in adherent granulosa cells (green, scale bar: 20 μm). White squares indicate distinctive cells and are zoomed. White arrows indicate membrane-distributed EGFR; (**b**) Portion of adherent granulosa cells with EGFR membrane distribution; (**c**) Expression of EGFR in granulosa cells was not significantly altered after 100 μg/mL fullerenol treatment for 2 h. Green, blue and red color indicate EGFR, nuclei staining and F-actin, respectively. At least three individual experiments were performed to detect EGFR levels.

The effect of fullerenol on EGFR distribution was investigated further. We found that EGFR (green) was commonly distributed in the cytoplasm and membrane in granulosa cells (Figure 7a, 0 µg/mL row indicated with a white arrow) in the control group, but was mainly distributed in the perinuclear region in fullerenol-treated cells (Figure 7a, 100 µg/mL row). Further, we found that in the control group, the percentage of granulosa cells with EGFR membrane distribution was 62.5% (85/136) while that in the treated group was reduced to 55.4% (67/121, Figure 7b). The result indicates that fullerenol induced the variation of EGFR cellular distribution. Additionally, EGFR expression in granulosa cells was not significantly altered after 100 μg/mL fullerenol treatment for 2 h (Figure 7c).

## 3. Discussion

**Figure 8:**
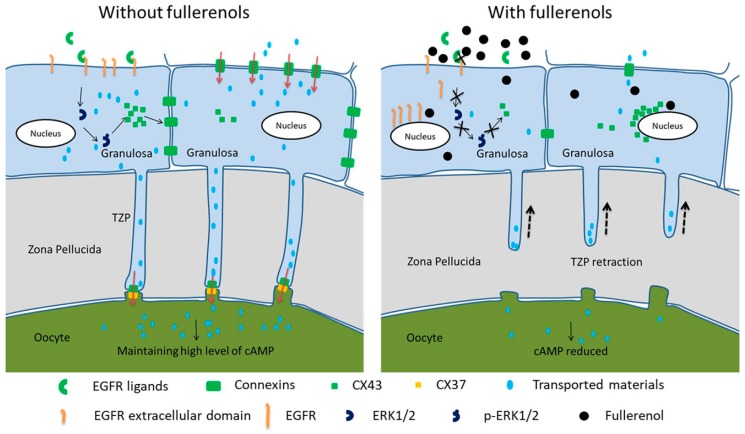
Schematic representation of effects of fullerenol nanoparticles on oocyte meiosis resumption. Fullerenol nanoparticles occluded extracellular domain of epidermal growth factor receptor (EGFR) to inhibit ligand-EGFR binding-mediated extracellular regulated kinase 1 and 2 (ERK1/2) activation. Then, connexin 43 (CX43) expression was downregulated, resulting in TZP retraction (dashed arrows) and reduction of gap junction-based material transport, which decreased cAMP in oocytes. Moreover, fullerenol nanoparticles caused perinuclear distribution of CX43 and EGFR, which further exacerbated the effects. Black arrows mean the signal transduction of EGFR mediated ERK1/2 activation and black cross (X) means fullerenol’s inhibitory effect. Orange arrows indicate material flow from granulosa cells to oocyte via TZPs.

Within ovarian follicles, interactions between the oocyte and its surrounding granulosa cells are extremely essential to generate fully competent oocyte [44]. TZPs and gap junctions provide important channels to persistently transport metabolites and signaling molecules to guarantee maturation of oocytes following a strict program [45]. Disrupting these connections leads to disorder and incomplete oocyte maturation, which potentially compromises oocyte quality. Hence, the females who might be exposed to fullerenol nanoparticles—including the workers on the production line and relevant personnel for the biomedical applications—should pay high attention to the potential adverse effects on the reproductive system in the case of long-term accumulation, which could increase the risk to health as discussed by Leso et al. [46]. In the present study, as summarized in Figure 8, we found that fullerenol nanoparticles occluded the extracellular domain of EGFR to inhibit ligand-EGFR binding-mediated ERK1/2 activation. This action subsequently downregulated CX43 expression. The loss of CX43 accelerated the retraction of TZPs, reduced the gap junction-based material transport, and reduced cAMP levels in oocytes. Moreover, we found that fullerenol nanoparticles induced CX43 and EGFR perinuclear distribution, which further exacerbated the effects by disabling their function.

Connexins have been reported to play essential roles in forming intercellular membrane channels in OGCs, highlighting the importance of these proteins in female reproductive health [47,48]. CX43 is a connexin, and follicles lacking this protein were arrested in the early preantral stages and produced incompetent oocytes [30]. Our results showed that CX43 protein and mRNA expression levels were downregulated in fullerenol-treated cells. In addition, an alteration in distribution from the membrane and cytoplasm to the perinuclear region in granulosa cells was observed (Figure 3). These results indicate that fullerenol nanoparticles played a role in disabling CX43 function.

CX43 expression and internalization in mammals are primarily correlated to direct or indirect activation of ERK1/2 [49]. p-ERK1/2 phosphorylates transmembrane CX43 rapidly by directly interacting with its intracellular domain, and then induces CX43 to internalize [50]. Furthermore, p-ERK1/2 is involved in regulating CX43 protein and gene expression. Therefore, we assayed ERK1/2 activation and found that the level of p-ERK1/2 in granulosa cells was sharply reduced by 100 μg/mL fullerenol treatment for 0 min to 2 h, and an extremely significant difference was observed at the 5 and 10 min time points compared with control. Interestingly, the p-ERK1/2 level was still lower in the treated cells than in the control during the 2 h treatment (Figure S4).

The EGFR/ERK1/2 signaling pathway plays a pivotal role in granulosa cells by adjusting oocyte maturation [42,51,52,53]. During in vitro maturation of cultured OGCs, ligand binding to EGFR on granulosa cells activated ERK1/2 [54,55]. Accumulating evidence suggests that nanoparticles can passively interact with cellular receptors, thereby specifically modulating signal transduction pathways by activation or inhibition [56,57]. In the present study, we found that fullerenol reduced the p-ERK1/2 level. Therefore, the effect of fullerenol on ERK1/2 activation via EGFR pathway was investigated by including free EGF or an antibody against the EGFR extracellular domain in the system. The results indicate that fullerenol and the antibody acted similarly in inhibiting EGFR-mediated ERK1/2 activation (Figure 5). Further, targeting the extracellular domain of EGFR in granulosa cells with the antibody revealed that the visible fluorescence was significantly reduced by fullerenol pretreatment (Figure 6). This further implies that fullerenol occluded the extracellular domain of EGFR to block ligand binding.

Thus, fullerenol possibly blocks the binding of ligands and EGFR to reduce ERK1/2 activation, which subsequently downregulates CX43 expression. Furthermore, p-ERK1/2 could directly interact with and phosphorylate the cytoplasmic domain of CX43, which could then be internalized [58,59]. In this study, internalization of CX43 corresponded to the reduction of p-ERK1/2. Further, we found that fullerenol obviously altered the distribution pattern of CX43 in granulosa cells. In the control cells, CX43 aggregated as spots and was randomly distributed in the cytoplasm, whereas it was mainly distributed in perinuclear region in treated cell. The percentage of cells with CX43 perinuclear distribution in the control and treated groups was 7.1% and 37.9%, respectively. An interesting phenomenon observed was that the internalized EGFR was similarly distributed in the perinuclear region of treated cells, but was distributed in the cytoplasm and membranes of control cells. This may have reduced the EGFR density in the membranes. Nanoparticles with a small size have been reported to likely accumulate in the perinuclear region after uptake [60,61,62]. However, whether these particles induce proteins on the cell membrane for internalization and distribution in the perinuclear region has not been ascertained. These results imply that fullerenol nanoparticles regulated the distribution pattern of the membrane protein. However, further studies are required to reveal the molecular mechanisms.

## 4. Materials and Methods

### 4.1. Preparation of Fullerenol Nanoparticles

Fullerenol nanoparticles were prepared as previously described [63,64]. Atomic force microscopy (AFM, Agilent 5500, Santa Clara, CA, USA) was used to monitor the particle size, as detailed in Figure S5. After ultrasonic treatment, a 2 mg/mL stock solution in M2 medium (Sigma-Aldrich, St. Louis, MO, USA) was diluted to the indicated concentrations just before use.

### 4.2. Dosage and Concentration Selection

Previous acute toxicity test in mice showed that the median lethal dose (LD_50_) of fullerenol was 386.1 mg/kg body weight. Hence, for the treatment in rats, the following graded doses were selected: 2.537, 5.074, and 25.370 mg/kg body weight. In the long-term preliminary experiment, fullerenol was dissolved in normal saline and administered to female rats via intravenous tail injections for 3 consecutive days every week. After 12 weeks, copulations between normal males and fullerenol-treated females were set, and the females were euthanized 18 days after mating. The uterus and fetus were collected from each rat (Figure S1) and obvious growth inhibition of the fetus was observed at the 25.370 mg/kg body weight dose, which produced a fullerenol blood concentration of approximately 396 μg/mL (the average blood volume of a rat is approximately 6.4 mL/100 g body weight). Therefore, the graded concentrations of fullerenol applied to the in vitro cultured cells were 1, 10, and 100 μg/mL. More significant differences were observed at the high concentration (100 μg/mL). Hence, this concentration was used to evaluate the potential effect of fullerenol nanoparticles on oocyte meiosis resumption.

### 4.3. Oocyte, Granulosa Cell Collection, and Culture

Female Sprague-Dawley (S-D) rats (body weight, 110 ± 10 g) were purchased from Laboratory Animal Center, Academy of Military Medical Science of People’s Liberation Army (Beijing, China). All animal experiments were approved by the Medical Ethics Committee of Peking Union Medical College (No. YZS20160315012, 15 March 2016). The rats were superovulated by intraperitoneal injection of 20 IU pregnant mare’s serum gonadotrophin (PMSG, P9970, Solarbio, Beijing, China) and 45–48 h later, GV-stage oocytes with intact surrounding granulosa cells were collected. After several washes, the OGCs were placed into Petri dishes with pre-warmed M2 medium in different groups with or without the test substances—fullerenol (indicated concentrations), EGF (20 pg/mL, Invitrogen, Carlsbad, CA, USA) and EGFR antibody (2 μg/mL, sc-31155, Santa Cruz Biotechnology, Dallas, TX, USA)—in 50 μL droplets covered with mineral oil (15 OGCs per droplet) and cultured in a humidified atmosphere of 5% CO_2_ in air at 37 °C. For GVBD detection, OGCs were transferred to phosphate-buffered saline (PBS) containing 1% hyaluronidase (H4272, Sigma-Aldrich, St. Louis, MO, USA) at the end of the 5-h culture to remove granulosa cells and then they were morphologically assessed using an optical microscope. The oocytes were removed from the freshly isolated OGCs using hyaluronidase, the granulosa cells were collected and washed in PBS and then routinely cultured in a chamber slide system (Nunc™ Lab-Tek™, Thermo Fisher Scientific, Waltham, MA, USA) in Dulbecco’s modified Eagle’s medium (DMEM)/F12 medium (Hyclone, Carlsbad, CA, USA) with 10% fetal bovine serum (FBS, Gibco, Carlsbad, CA, USA) for 4 days to attach. The fullerenol concentrations applied to the adherent granulosa cells were the same as those used on the OGCs.

### 4.4. Confocal Image Processing

For immunofluorescent staining, OGCs or adherent granulosa cells were fixed with 4% paraformaldehyde (PFA) in PBS for at least for 30 min. After permeabilization and blocking (PBS containing 10% goat serum, 0.3 M glycine, 1% bovine serum albumin (BSA) and 0.1% Tween-20) for 2 h, the cells were incubated with primary antibodies—CX43 (#3512, CST, Danvers, MA, USA), cAMP (sc-73761, Santa Cruz Biotechnology, Dallas, TX, USA), follicle-stimulating hormone receptor (FSHR, BS2618, Bioworld Technology, St Louis Park, MN, USA) and EGFR (sc-31155, Santa Cruz Biotechnology, Dallas, TX, USA)—in blocking buffer solution (PBS containing 1% BSA, 0.1% Tween-20, and 0.01% Triton X-100) for 2 h. After several washes with PBS containing 0.1% Tween-20, the samples were labeled with DyLight^®^ 488-conjugated anti-rabbit IgG (ab96899, Abcam, Cambridge, UK), Alexa 594-conjugated anti-mouse IgG (A-11005, Thermo Fisher Scientific, Waltham, MA, USA), DyLight^®^ 488-conjugated rabbit anti-goat IgG (A23230, Abbkine, Redlands, CA, USA), or Alexa Fluor^®^ 488-conjugated goat anti-mouse IgG (ab150117, Abcam, Cambridge, UK) secondary antibodies for 1 h in the dark at room temperature (20–26 °C). After several washes, the cells were incubated with or without rhodamine phalloidin for 20 min to label the F-actin cytoskeleton and ProLong mounting medium (ProLong Gold, Molecular Probes, Eugene, OR, USA) and 4,6-diamidino-2-phenyl-indole (DAPI) was used to stain the nuclei. Finally, the samples were examined using a laser scanning confocal microscope (Nikon Ti-E imaging system, Tokyo, Japan).

### 4.5. Anti-EGFR Extracellular Domain Targeted Antibody Binding Capacity Analysis

Standard immunofluorescent staining procedures as described above were used with modifications. Briefly, freshly isolated OGCs were rapidly fixed and exposed to 100 μg/mL fullerenol in PBS for 10 min at room temperature (20–26 °C), and then they were gently transferred to PBS to remove the fullerenol buffer before incubation in anti-EGFR primary antibody solution.

### 4.6. Immunoblotting Analysis

OGCs were collected, washed in PBS, and then treated with 1% hyaluronidase to release the granulosa cells from the oocytes. Then, the oocytes and granulosa cells were washed several times in cold PBS and then separately placed into 1× sodium dodecyl sulfate (SDS) buffer, and heated for 10 min at 100 °C. After centrifugation (10,000× *g*, 15 min), the proteins were separated using SDS-polyacrylamide gel electrophoresis (PAGE) and then transferred to polyvinylidene fluoride (PVDF) membranes. The membranes were subsequently blocked in Tris-buffered saline plus Tween (TBST) containing 5% skim milk for 1 h at room temperature, followed by incubation overnight at 4 °C with rabbit polyclonal anti-CX43, rabbit polyclonal anti-CX37 (ab181701; Abcam, Cambridge, UK), rabbit monoclonal anti-phosphorylated-ERK 1 and 2 (p-ERK1/2, #4370, CST, Danvers, MA, USA), rabbit monoclonal anti-ERK1/2 (#4695, CST, Danvers, MA, USA), mouse monoclonal anti-EGFR (sc-365829, Santa Cruz Biotechnology, Dallas, TX, USA), or mouse monoclonal anti-glyceraldehyde 3-phosphate dehydrogenase (GAPDH, TA-08; Zhongshan Golden Bridge Biotechnology, Beijing, China) antibodies. After four washes in TBST for 10 min each, the membranes were incubated with horseradish (HRP)-conjugated goat anti-mouse (ZB-2305, Zhongshan Golden Bridge Biotechnology, Beijing China) or HRP-conjugated goat anti-rabbit IgG (ZB-2301, Zhongshan Golden Bridge Biotechnology, Beijing, China) antibody for 2 h at room temperature. Finally, the membranes were processed using the Tanon 5200 multi chemiluminescent imaging system (Tanon, Shanghai, China).

### 4.7. Reverse Transcription-Polymerase Chain Reaction (RT-PCR) Analysis

Total mRNA from granulosa cells was isolated using Trizol reagent (Invitrogen, Carlsbad, CA, USA). Standard polymerase chain reaction (PCR) analysis was performed to assess changes in the levels of mRNAs encoding CX43 and GAPDH. The primers for *CX43* and *GAPDH* and operating procedures are listed in the additional files (Tables S1 and S2).

### 4.8. Statistical Analysis

The investigator was blinded to each treatment while measuring the TZP parameters and fluorescence imaging of CX43, EGFR, and cAMP. The results of the western blot and fluorescent intensity assays of CX43, cAMP and the nuclei were analyzed using ImageJ (version 1.37, Wayne Rasband, National Institutes of Health (NIH), Bethesda, MD, USA). The data were analyzed using a one-way analysis of variance (ANOVA) with the Prism 5.01 GraphPad for Windows (GraphPad Software, San Diego, CA, USA). A two-tailed *t*-test was used when independent two percentages were compared. Statistical significance was accepted for *p*-values < 0.05 or when the test statistic was greater than the critical value.

## 5. Conclusions

Fullerenol nanoparticles occluded ligand-EGFR binding to inhibit EGFR-mediated ERK1/2 activation. Reduced p-ERK1/2 downregulated CX43 expression in granulosa cells and then accelerated TZP retraction from the oocytes. The accelerated retraction reduced cAMP levels in the oocytes, which resulted in premature GVBD. Therefore, the strict program of oocyte maturation was disrupted, which ultimately reduced the oocyte quality.

## Figures and Tables

**Table 1:** Effect of fullerenol on oocyte germinal vesicle breakdown (GVBD).

Groups (μg/mL)	No. of GV Stage Oocytes	GVBD (%)
0	264	212 (80.3%)
1	212	186 (87.7%) *
10	266	239 (89.8%) *
100	180	162 (90.0%) *

After isolation, OGCs were cultured in M2 medium with different concentrations of fullerenol (0, 1, 10, and 100 μg/mL) for 5 h and then GVBD in each group was morphologically examined after removing granulosa cells with hyaluronidase. * Indicates the test statistic was greater than the critical value when the indicated group and the 0 μg/mL group were compared.

## Supplementary Materials

Supplementary materials can be found at http://www.mdpi.com/1422-0067/19/3/699/s1.

## Abbreviations

OGCs	oocyte-granulosa cell complexes
EGFR	epidermal growth factor receptor
ERK1/2	extracellular signal-regulated kinase 1 and 2
CX43	connexin 43
TZPs	transzonal projections
cAMP	cyclic adenosine monophosphate
GV	germinal vesicle
LH	luteinizing hormone
GVBD	germinal vesicle breakdown
p-ERK1/2	phosphorylated extracellular signal-regulated kinase 1 and 2
AFM	atomic force microscopy
LD_50_	median lethal dose
PMSG	pregnant mare’s serum gonadotrophin
EGF	epidermal growth factor
PBS	phosphate-buffered saline
FBS	fetal bovine serum
DMEM	Dulbecco’s modified Eagle’s medium
PFA	paraformaldehyde
BSA	bovine serum albumin
FSHR	follicle stimulating hormone receptor
DAPI	4,6-diamidino-2-phenyl-indole
SDS	sodium dodecyl sulfate
PAGE	polyacrylamide gel electrophoresis
PVDF	polyvinylidene fluoride
TBST	Tris-buffered saline plus Tween
RT-PCR	reverse transcription-polymerase chain reaction
PCR	polymerase chain reaction
