# Supplementary material for: Effects of Fullerenol Nanoparticles on Rat Oocyte Meiosis Resumption
Source: Int J Mol Sci. 2018 Mar 1;19(3):699. doi: 10.3390/ijms19030699 (PMC5877560; doi:10.3390/ijms19030699)
Supplement: Supplementary file 1 [file ijms-19-00699-s001.pdf]

International Journal of
*Molecular Sciences*

MDPI

# Supplementary Materials: Effects of Fullerenol Nanoparticles on Rat Oocyte Meiosis Resumption

**Runhong Lei, Xue Bai, Yanan Chang, Juan Li, Yanxia Qin, Weihong Gu, Kui Chen, Shibo Xia, Jiaxin Zhang, Zhenbo Wang,* and Gengmei Xing***

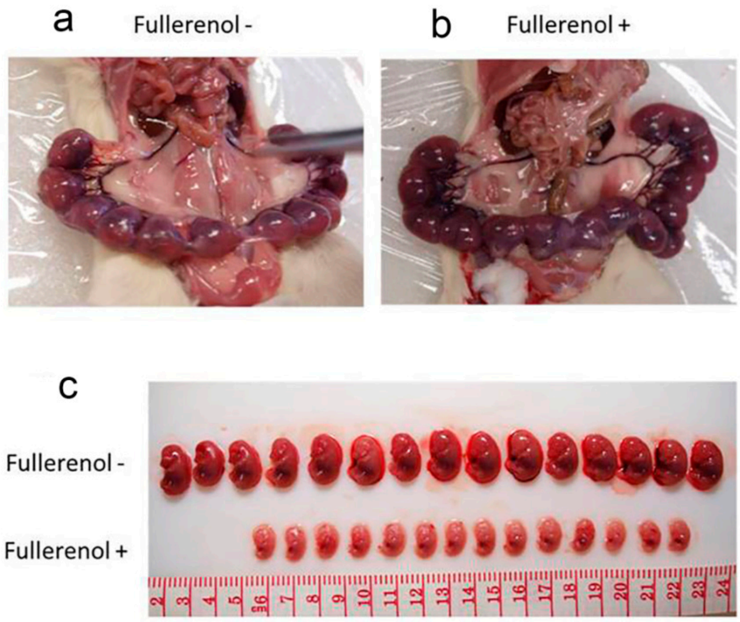


**Figure S1**. Uteri and fetuses from female rats treated with or without fullerenol. Fullerenol was dissolved in normal saline (25.370 mg/kg body weight) and administered to female rats by tail intravenous injection for 3 consecutive days every week. After a 12-week treatment, copulations between normal males and fullerenol-treated females were initiated. Females were euthanized 18 days after treatment, and the uteri were collected. Fetal development degree was recorded. Uterus of (**a**) control and (**b**) fullerenol-treated rats. Darker color was observed after fullerenol treatment. (**c**) Fetal development in both groups. Smaller fetus was observed after fullerenol treatment (n = 3 in each group).

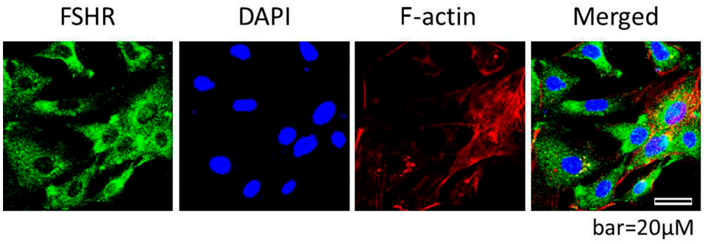


**Figure S2.** Immunofluorescent staining of follicle-stimulating hormone receptor (FSHR) in granulosa cells. Freshly isolated granulosa cells were routinely cultured for 4 days to attach and then fixed in paraformaldehyde (PFA). Identification with specific cell type marker FSHR indicated the high purity of granulosa cells (green, scale bar: 20 μm). Blue and red color indicate nuclear staining and F-actin skeleton, respectively. Imaging was repeated three times with similar results.

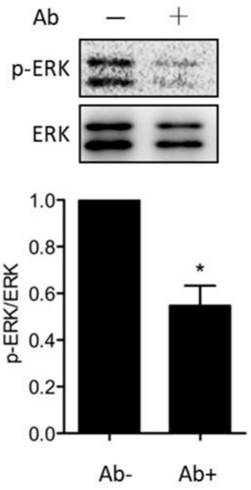


**Figure S3.** Extracellular domain-targeted anti-epidermal growth factor receptor (EGFR) antibody reduced phosphorylated-extracellular signal-regulated kinase 1 and 2 (p-ERK1/2) activation in granulosa cells. Freshly isolated oocyte-granulosa cell complexes (OGCs) were cultured in M2 medium with or without 2 μg/mL antibody for 2 h, and then granulosa cells were harvested after hyaluronidase treatment for immunoblotting analysis. Ab, extracellular domain-targeted anti-EGFR antibody. Data are shown as mean ± standard deviation (SD) from three replicates. Typical images are presented.

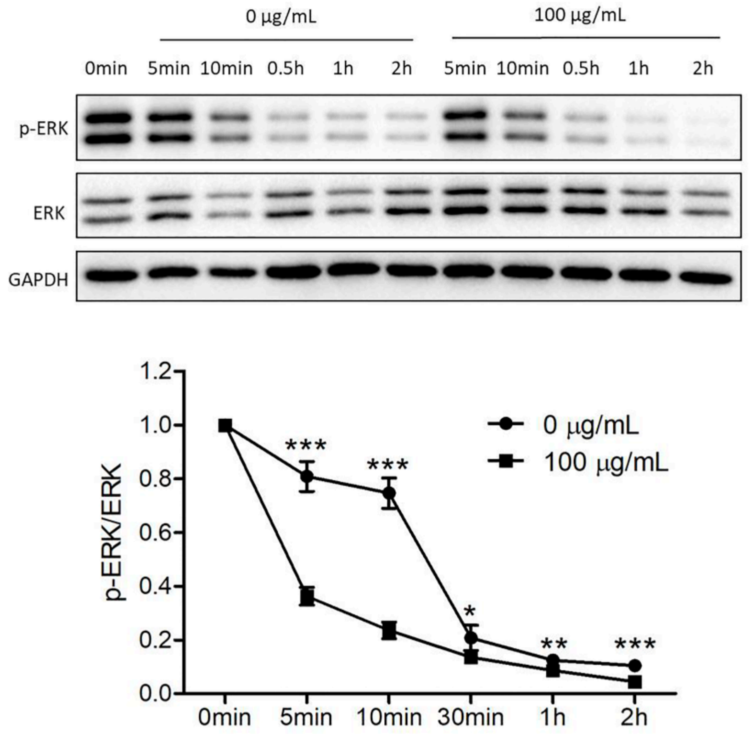


**Figure S4.** Time-course of extracellular signal-regulated kinase 1 and 2 (ERK1/2) activation in granulosa cells after fullerenol treatment. Freshly isolated oocyte-granulosa cell complexes (OGCs) were cultured in M2 medium with or without 100 μg/mL fullerenol for different times, and then granulosa cells were harvested after removing oocytes using hyaluronidase treatment for immunoblotting analysis. Data are shown as mean ± standard deviation (SD) from three replicates. Typical images are presented.

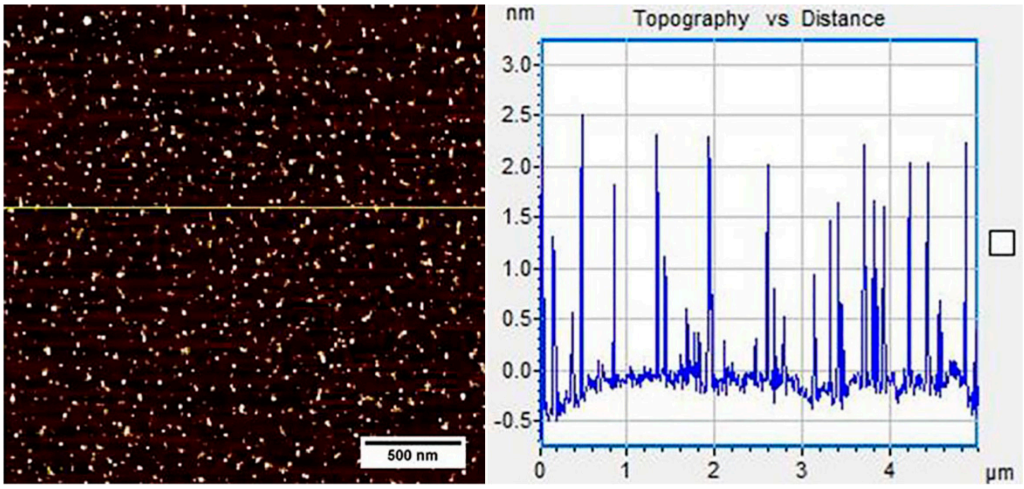


**Figure S5.** Characterization of fullerenol nanoparticles using atomic force microscopy (AFM). Fullerenol nanoparticles were dissolved in phosphate-buffered saline (PBS) and ultrasonically processed. Newly cleaved mica was pretreated with 0.1 M magnesium chloride ($MgCl_2$), rinsed with deionized water and then dried before nanoparticles deposition. AFM (Agilent 5500, Santa Clara, CA, USA) imaging was performed under air condition and AC mode (tapping mode). High-quality (HQ): NSC15/ALBS probes with nominal spring constant of K = 40 N/m from Mikromasch probes were used. Settings: amplitude, 3–6 V; speed, 0.8 ln/s; scan size, 10 × 10 µm; and range, 1.5 µm. Images were processed off-line using inherent PicoImage software.

**Table S1.** RT-PCR Primers of *CX43* and *GAPDH*

| Gene name | Primers |
|---|---|
| *CX43* | Forward: 5′-CTCACGTCCCACGGAGAAAA-3′,<br>Reverse: 5′-CGCGATCCTTAACGCCTTTG-3′ |
| *GAPDH* | Forward: 5′-TACCCACGGCAAGTTCAACG-3′,<br>Reverse: 5′-CACCAGCATCACCCCATTTG-3′ |

**Table S2.** RT-PCR protocol

| **Reverse Transcription** | |
|---|---|
| 1. Prepare the following mixture in each tube: | |
| Total RNA | 3 µg |
| Olig dT | 2 µl |
| DEPC $H_2O$ | to 14 µl |
| 2. Incubate the samples at 70°C for 5 min and then on ice for at least 1 min. | |
| 3. Prepare reaction master mixture. For each reaction: | |
| RNase inhibitor | 0.7 µl |
| M-MLV buffer | 5 µl |
| M-MLV | 1 µl |
| dNTP | 1 µl |
| dd$H_2O$ | to 25 µl |
| 4. Incubate the tubes at 37°C for 90 min, heat inactivate at 70°C for 5 min, and then chill on ice. | |
| 5. Store the cDNA at -20°C until use for real-time PCR. | |
| **Real-time PCR** | |
| 1. Prepare the following mixture in each tube: | |
| cDNA | 2 µl |
| Primer (forward and reverse) | 1 µl + 1 µl |

| | |
|---|---|
| dNTP | 1 μl |
| Buffer | 5 μl |
| DNA polymerase | 0.5 μl |
| DEPC $H_2O$ | to 25 μl |

2. Set up the experiment and the following PCR program as follow.

a. 95°C 3 min, 1 cycle

b. 95 °C 30 s -> 55 °C 30 s -> 72 °C 1 min, 34 cycles

c. 72°C 5 min, 1 cycle

3. After PCR is finished, remove the tubes from the machine. The PCR specificity is examined by 1% agarose gel using 5 μl from each reaction.
